# The Impact of the COVID-19 Pandemic on Ambient Air Quality in China: A Quasi-Difference-in-Difference Approach

**DOI:** 10.3390/ijerph18073404

**Published:** 2021-03-25

**Authors:** Tuo Zhang, Maogang Tang

**Affiliations:** 1 Graduate School of Economics, Kyoto University, Yoshidahonmachi, Sakyo Ward, Kyoto 606-8501, Japan; ZhangTuoDr@hotmail.com; 2 School of Business, East China University of Science and Technology, 130 Meilong Road, Shanghai 200237, China

**Keywords:** COVID-19, ambient air quality, air pollution emissions, quasi-difference-in-difference, Q51, Q52, Q53

## Abstract

The novel coronavirus (COVID-19) pandemic has provided a distinct opportunity to explore the mechanisms by which human activities affect air quality and pollution emissions. We conduct a quasi-difference-in-differences (DID) analysis of the impacts of lockdown measures on air pollution during the first wave of the COVID-19 pandemic in China. Our study covers 367 cities from the beginning of the lockdown on 23 January 2020 until April 22, two weeks after the lockdown in the epicenter was lifted. Static and dynamic analysis of the average treatment effects on the treated is conducted for the air quality index (AQI) and six criteria pollutants. The results indicate that, first, on average, the AQI decreased by about 7%. However, it was still over the threshold set by the World Health Organization. Second, we detect heterogeneous changes in the level of different pollutants, which suggests heterogeneous impacts of the lockdown on human activities: carbon monoxide (CO) had the biggest drop, about 30%, and nitrogen dioxide (NO_2_) had the second-biggest drop, 20%. In contrast, ozone (O_3_) increased by 3.74% due to the changes in the NO_x_/VOCs caused by the decrease in NO_x_, the decrease of O_3_ titration, and particulate matter concentration. Third, air pollution levels rebounded immediately after the number of infections dropped, which indicates a swift recovery of human activities. This study provides insights into the implementation of environmental policies in China and other developing countries.

## 1. Introduction

At the end of 2019, an unusual coronavirus disease, eventually named COVID-19, was identified in Wuhan, China [1]. To curb its spread, the Chinese government enacted lockdown measures in the epicenter on 23 January 2020. The lockdown was expanded to the rest of the country soon after [2]. Non-essential businesses were closed, and residents were quarantined at home to cut off the viral transmission [3]. The drastic lockdown worked successfully [4,5], and it took 76 days for the epicenter to reopen.

These measures significantly reduced industrial, business, and residential activities [6]. One of the most concerning aspects is that energy consumption is reduced by the drastic lockdown measures and the cessation of human activities [7]. For instance, Wang, et al. [8] suggest that the fossil fuel-related CO_2_ emissions in China decreased by 18.7% YOY in the first quarter of 2020. Since ambient air quality is closely related to energy consumption, prior studies found that the air quality is improved dramatically during lockdown [9]. He, et al. [10] found that the operating vent numbers of NOx decreased by 24.68% in China during the lockdown period, which would reduce the NOx concentration by 9.54 ± 6.00.

The pandemic provided a distinct opportunity to examine the mechanisms and ways in which human activities affect air quality and pollution emissions. Moreover, in-depth research through a quasi-experiment of nature is worth conducting [11]. However, previous research has some limitations. First, most recent findings are based on descriptive-comparative methods, and the lack of proper identification strategies threatens the validity of their results; for instance, the direct comparison of air quality before and after lockdown overestimates its impacts, as seasonal trends are ignored. Second, most previous studies only cover a short period, which limits comprehensive interpretation not only on the shrinkage but also on the rebound effect [10]. In this case, the rebound effect is of more concern since it captures the economic recovery from the deadly shock of COVID-19. Third, the results of previous research are mostly static and lack a dynamic analysis.

Therefore, we adopt a quasi-difference-in-difference (quasi-DID) approach, which enables the comparison of air quality between the epidemical period in 2020 and Chinese New Year’s leave in 2019, to estimate the net impact of the lockdown during the first peak of COVID-19. Moreover, through dynamic analysis, we identify the varying impact of the lockdown on air quality, which facilitates our understanding of human responses to the epidemic.

Our results suggest that, first, on average, the air quality index (AQI) decreased by about 7%. Although our results indicate immense improvements, the air quality was still above the threshold set by the World Health Organization (WHO) and Chinese health standards. Second, we detect significant heterogeneous impacts on different pollutants. Carbon monoxide (CO) had the highest biggest drop, about 30%, and nitrogen dioxide (NO_2_) had the second-highest drop, about 20%. In contrast, ozone (O_3_) increased by 3.74% due to the changes in the NO_x_/VOCs caused by the decrease in NO_x_, the decrease of O_3_ titration, and the decrease of PM_2.5_ concentration. Third, although the AQI fell steeply after the lockdown, it increased immediately after the number of novel infections dropped, which indicates a swift economic recovery. Besides, we document preliminary cues of the rebound effect immediately after the lifting of lockdown measures in Wuhan.

This study’s contribution to the literature is two-fold. First, compared with the recent studies in this field, our period covers the whole lockdown period, from the beginning of the lockdown on January 23 to two weeks after the lift of lockdown in Wuhan. Therefore, it enables us to not only identify the shrinkage but also study the rebound of air pollution and human activities, which is more relevant to current opening-up processes in most regions. To the best of our knowledge, this is the first study that identifies the dynamic impacts of lockdown measures on the environment. Second, this study also contributes to future environmental policy measures. Although the temporary shutdown of pollution-intensive plants has become a common practice during periods of extreme air pollution, the impact of such emergency measures is still unclear. Our research sheds light on the mechanisms of human activities affecting air quality and pollution emissions.

The remainder of this paper is organized as follows: Section 2 provides information on the data sources and empirical methodology. Section 3 presents the average effect of the lockdown on the air quality in China. Section 4 reports the dynamic patterns of the effects of the COVID-19 lockdown. Finally, Section 5 summarizes this study and discusses its limitations.

## 2. Data and Empirical Methodology

### 2.1. Datasets

In this study, we combine three datasets: hourly real-time reports of air pollutants, daily historical meteorological information, and pandemic data. All three datasets are at the prefecture and county levels and cover 367 cities in China.

Air quality data were collected from the China National Urban Air Quality Real-time Publishing Platform sponsored by the China National Environmental Monitoring Center. The platform reports the concentrations of six air pollutants—SO_2_, NO_2_, CO, O_3_, PM_10_, and PM_2.5_ (in micrograms [µg] per cubic meter under standard conditions)—as well as the aggregate AQI based on *the Chinese Technical Regulation on Ambient Air Quality Index*. Its wide coverage facilitates our investigation of the lockdown’s impact on different human activities. For example, NO_2_ is an effective way to track transportation in urban areas [12], while SO_2_ is mostly caused by flue gas of coal-fired boilers [13]. Notably, air quality monitoring stations are always located within urban areas, especially for prefecture-level cities [14]. Therefore, the pollution data largely represent air quality in the downtown areas of cities.

We collected prefectural infection data from a public GitHub repository and crosschecked the data against official daily reports by the National Health and Family Planning Commission. This dataset contains daily cumulative confirmed cases, cumulative death toll, and cumulative recovered cases for each infected city since 1 December 2019, when the first case was traced back to Wuhan.

Meteorological conditions also influence ambient air quality [15,16,17]. We took daily meteorological information from the website of the China Meteorological Data Service Center. Data reported by meteorological stations located in the city downtowns were chosen to match the collected air quality information. Thus, in our analysis, we could control for meteorological conditions, including temperature [18], precipitation [19], and wind [20], which affect air pollutants transmissions.

### 2.2. Quasi-DID Identification

#### 2.2.1. Identification Strategy

**Figure 1:**
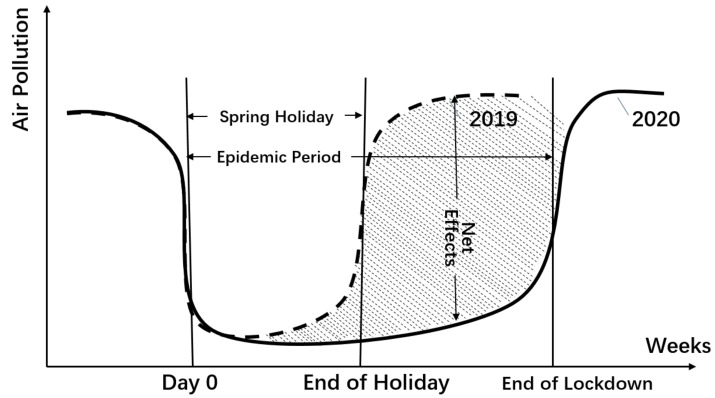
Illustration of an identification strategy. Note: This figure illustrates the quasi-DID approach in this study. Day zero in 2019 is set as the beginning of Chinese New Year’s leave, February 5, while day zero in 2020 is set as the beginning of the lockdown period for most Hubei cities, January 23. We choose 22 April 2020, as the end of our research period in 2020, two weeks after Wuhan lifted its lockdown and resumed transportation conditionally on April 8.

Previous studies have used various events to explore exogenous shocks on air quality. For example, Chiquetto, et al. [21] studied the impact of a sudden truck driver strike in Sao Paulo on urban air pollution. Li, et al. [22] took the suspended production of heavy-polluting factories during the 2008 Beijing Summer Olympics as an environmental event to study its impact on outpatient visits for asthma owing to the improvement of air quality. Additionally, Feng, et al. [23] conducted an event study on the environmental impact of the Chinese Spring Festival. In this vein, we build a quasi-DID model to identify the causal relationship between air pollution and the lockdown imposed under the COVID-19 state of emergency in China. As illustrated in Figure 1, if the influence of meteorological conditions is not considered, there is a clear reversal in air quality during the Chinese New Year holiday [23,24]. As the new year approaches, factories shut down and release their workers so they can travel home and spend time with their families during the Spring Festival (which was 24–30 January 2020) [25]. Besides, most industrial plants remain closed until the end of the holiday [26].

The lockdown measures induced by the COVID-19 pandemic have functioned similarly to the usual New Year holiday period [27]. Residents are required to stay at home and are only allowed to visit nearby grocery stores. Most factories are temporarily shut down [4]. Therefore, non-essential industrial activities are restricted. Highways, as well as major carriageways, are completely blocked. Only vehicles with special permissions can travel across jurisdictions [28].

Therefore, it is feasible to compare the air quality between the 2020 lockdown period and the 2019 Chinese New Year’s holiday to estimate the net impact of the COVID-19 lockdown. Day zero in 2019 is set as the beginning of Chinese New Year’s leave, February 5, while day zero in 2020 is set as the beginning of the lockdown period for most Hubei cities, January 23. We choose April 22 as the end of our research period, two weeks after Wuhan lifted its lockdown and resumed transportation conditionally on April 8.

As shown in Figure 1, we can calculate the daily impact, which is given by the concentration of air pollutants in 2019 minus that in 2020. We can also identify the dynamic impacts day by day. Besides, the shaded area, as the integral of impact over time, represents the aggregated impact of the COVID-19 lockdown on air quality. Compared to the single difference model used in other studies, such as Li, et al. [29], our DID approach can eliminate the impact of the New Year holiday; hence, it is more accurate in estimating the average treatment effects on the treated.

#### 2.2.2. The Average Effect on Air Pollution

Based on the above analysis, we evaluate the treatment impact of the lockdown on air pollution with the following DID regression equation:

(1) $$y_{it}=\gamma Treat_{i}\times Post_{t}+\beta_{1}Treat_{i}+\beta_{2}Post_{t}+\Gamma X_{it}+\eta_{i}+\delta_{t}+\varepsilon_{it}$$

where the dependent variable *y_it_* is the proxy for air quality. The dummy variable *Treat* takes “1” for observations in 2020 and “0” for those in 2019. The dummy variable *Post* takes “1” for periods after January 23 in 2020 and “1” after February 5 in 2019. The term *η_i_* captures the prefectural fixed effects, *δ_t_* is the time fixed effects, and *ε_it_* is the random error term.

Besides, we control for a full set of daily meteorological variates *X_it_* in Equation (1) following previous research [30,31]. *X_it_* contains the highest and lowest temperatures, the Beaufort scale of predominant winds in 24 h, and a dummy for rainy days.

The coefficient obtained by the first difference before and after the 2020 lockdown is *γ* + *β*_2_. The coefficient obtained by the first difference before and after the 2019 Spring Festival is *β*_2_. We differentiate the two distinct results again, so *γ* captures the net effect of the lockdown measures after the COVID-19 outbreak.

#### 2.2.3. Dynamic Impacts on Air Pollution

We investigate the dynamic evolution of the impacts on air pollution by using the following equation:

(2) $$y_{it}=\overset{n}{\underset{t=1}{\sum }}\gamma_{t}Treat_{i}\times Post_{t}+\beta_{1}Treat_{i}+\beta_{2}Post_{t}+\Gamma X_{it}+\eta_{i}+\varepsilon_{it}$$

where *Post* is the dummy variable for a specific period after day zero. In our analysis framework, *Post_t_* is defined as the *t^th^* week after the beginning of the lockdown. Therefore, the coefficient *γ_t_* captures the net effects during its corresponding week *t*.

### 2.3. Summary Statistics

We report the summary statistics of the urban ambient AQI for the two periods in Table 1. The observations in the control and treatment groups are divided into pre-periods before the event day and post-periods after the event day.

Panel A reports the summary statistics for the control group in 2019. The average AQI for the whole period, pre-period, and post-period are 77.32, 92.40, and 71.55, respectively. Compared to the pre-period, the average AQI decreased by 20.85, or 22.56%. We find a similar pattern in the change in the median. The medians of the three periods are 64.21, 78.94, and 61.08, respectively. Moreover, we find a sharp decrease of 17.86, or 22.62%, in the median of the AQI. The decrease is likely attributed to two factors: the seasonal change caused by meteorological conditions and socioeconomic factors such as the spring festival.

Panel B reports the summary statistics of the control group in 2020. The decrease recurs in that year. For example, the average AQI for the whole period, pre-period, and post-period are 67.86, 90.25, and 62.32, respectively. Compared with the pre-period, the average AQI decreased by 27.93, or 30.95%. The medians of the three periods are 56.58, 72.71, and 54.25, respectively. Besides, we find a sharp decrease of 18.45 or 25.37% in the median of the AQI. The decrease is likely attributed to two factors: the seasonal change caused by meteorological conditions and socio-economic factors such as the lockdown measures induced by the COVID-19 pandemic.

As for the quasi-DID design, we can roughly estimate the impacts of the COVID-19 lockdown on the AQI by subtracting the AQI decrease in 2020 from the decrease in 2019. For instance, the average treatment effect is roughly 7.07 for the AQI if meteorological conditions stay the same in both years. Although the decline is significant, the AQI is still above the healthy level recommended by the WHO (World Health Organization (2 May 2018), *Ambient (outdoor) air pollution*, from https://www.who.int/news-room/fact-sheets/detail/ambient-(outdoor)-air-quality-and-health (accessed on 11 March 2021), and outdoor air quality is still unhealthy according to environmental non-government organizations [32].

Column (1) of Panel C reports the comparisons of the group mean and the t-test results for the AQI. We can see that in the single difference design, the AQI decreases significantly in both years. However, the gap grew by 7.07 in 2020, which is 33.90% less than in 2019.

We also report comparisons of all types of air pollutants to obtain an integrated overview of the impacts. Five out of the six pollutants significantly decreased after the event day, except for O_3_. Fine atmospheric particulate matter PM_2.5_ and PM_10_ experienced the greatest drop. The levels of primary pollutants SO_2_, NO_2_, and CO also declined, confirmed by the pollution monitoring satellites of the National Aeronautics and Space Administration and the European Space Agency. The increase of O_3_ can mainly be attributed to the seasonal change of ultraviolet (UV) rays in solar radiation, which is a photo catalyst for the generation of O_3_ particles. Panel C also shows that the primary air pollutant during the COVID-19 pandemic is PM_2.5_, whose levels are nearly twice as high as the annual limits recommended by the WHO. Other pollutants, such as NO_2_ and SO_2,_ are well below their healthy levels.

**Figure 2:**
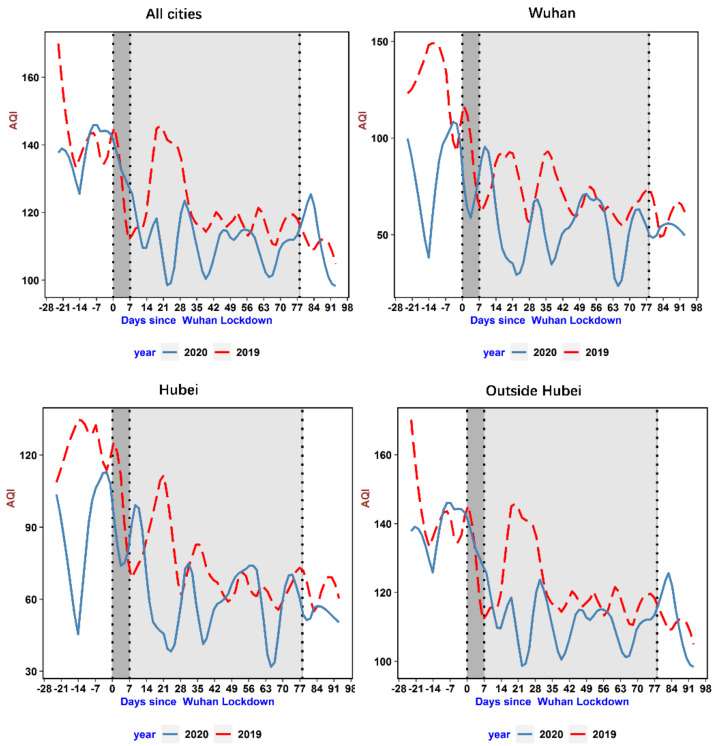
The time-varying patterns of the Air Quality Index (AQI) for different regions. Note: The daily average AQI is the average of the hourly AQI during the day. AQI is a simple, unitless index for reporting air quality and indicates the quality of the air and its health effects. Our sample covers 367 prefecture and county-level cities in China. The sample period for 2019 is from 14 January 2019to 9 May 2019, and the sample period for 2020 is from 1 January 2020 to 22 April 2020. The event day of 2020 is defined as January 23 (the date when the Wuhan lockdown was enacted), while the event day of 2019 is defined as February 5, one day before the Chinese New Year’s Eve. The red line shows the AQI variation for the sample period in 2020, while the blue line displays that for 2019. The 2019 Spring Festival is shown in dark grey, and the 2020 lockdown period is in light grey.

**Figure 3:**
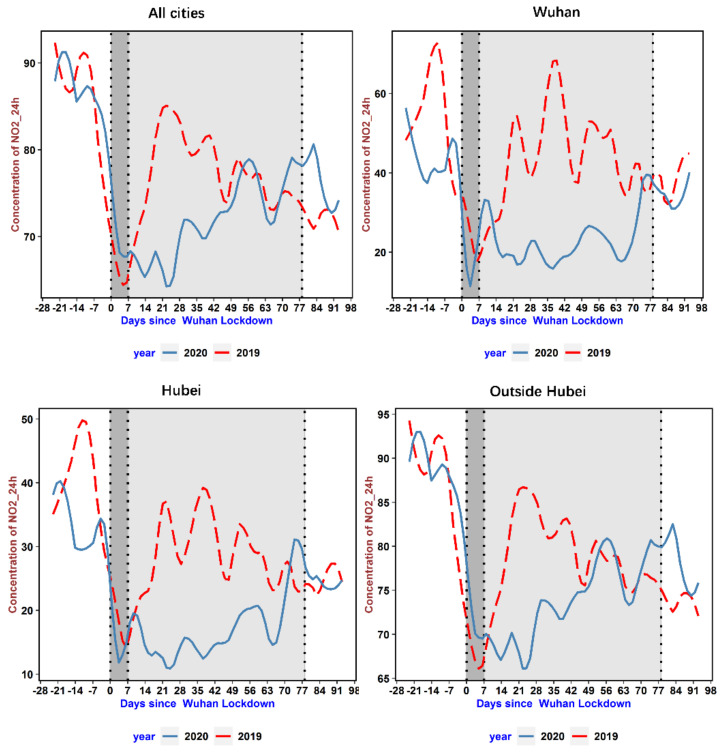
The time-varying patterns of NO_2_ for different regions. Note: The daily average of NO_2_ is the average of hourly NO_2_ levels during the day. The figure elements are the same as in Figure 2.

**Figure 4:**
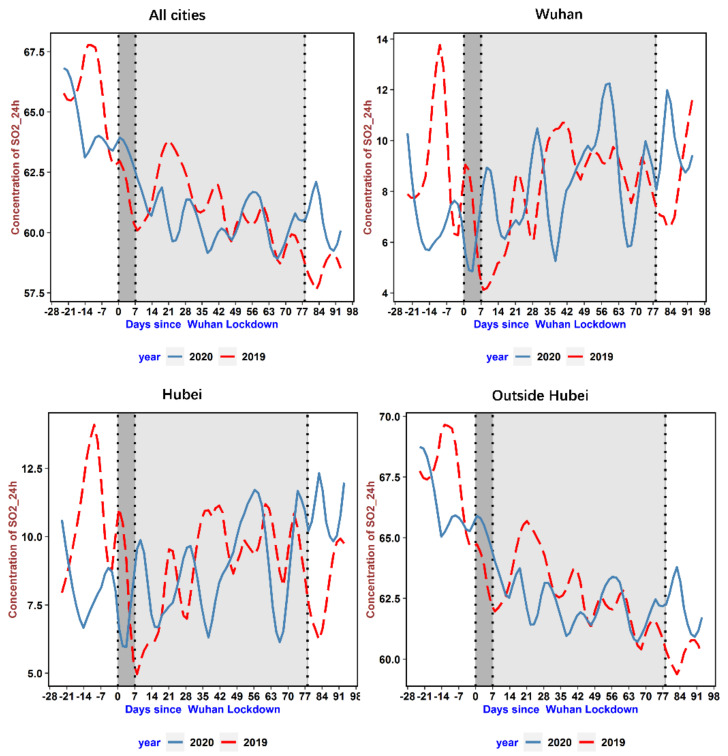
The time-varying patterns of SO_2_ for different regions. Note: The average daily SO_2_ is the average of hourly SO_2_ levels during the day. The figure elements are the same as in Figure 2.

We illustrate the time-varying patterns of the AQI and NO_2_ levels for regions of varying epidemic severity in Figure 2 and Figure 3, respectively. To show the impact of the COVID-19 lockdown on air pollution, the total sample is classified into four groups based on their epidemic severity, from highest severity to lowest: Wuhan city, cities inside Hubei Province, cities outside Hubei Province, and the full sample. The patterns for the AQI and NO_2_ levels are quite similar except that the changes in NO_2_ levels are typical. In the epicenter, Wuhan, we see a steep drop immediately after the lockdown. The pollution level stayed at its background concentration rate for nearly 12 weeks. The background concentration rate can be used to track fundamental human activities that were not affected by lockdown measures, for example, the transportation of daily necessities. Moreover, after the lockdown was lifted, the concentration gradually increased and returned to its normal level, just as it was in 2019. The pattern of pollution experienced in the cities in Hubei Province is similar to that in Wuhan. However, for the average city in China, the concentration of pollutants bounced back to normal levels around seven weeks after the event day, which is much quicker than in the epicenter. Figure 4 depicts the time-varying patterns of SO_2_, which shows that SO_2_ emissions instead increased when the lockdown was implemented. This SO_2_ emissions trend echoes that of 2019, but after about three weeks from the date of lockdown implementation, SO_2_ emissions were slightly lower than those in the same period in 2019. After economic activities resumed, SO_2_ emissions were higher than those in the same period in 2019 due to increased industrial production.

The parallel trend assumption is essential for the counterfactual setting in the DID approach [33]. All these figures show roughly similar trends of air quality change before the event day, which validates the parallel trend assumption.

## 3. The Average Effect on Air Quality

We begin by estimating the average effect of the COVID-19 lockdown on the daily AQI, and we estimate the results of Equation (1) with the AQI as the dependent variable. Alternative sets of control variates are reported in Table 2. Column (1) reports a model with no control on meteorological variates. The average net impact of the COVID-19 lockdown on the AQI is −7.125. This result is similar to our estimates in Table 1.

Unfavorable weather conditions lead to an increase in the level of air pollutants, even when emissions remain unchanged [34,35]. For example, high wind speeds lead to more dispersion of particulates [36]. Furthermore, the effect of wind speed on air quality is continuous, and a given day’s wind speed may affect air quality for several days. Therefore, in Column (2), we control the Beaufort scale of predominant winds in the current day and the past four days. In previous studies, only the wind on a particular day was analyzed [30,31]. We find that the wind scale of a particular day has little impact because the diffusion of pollutants takes time. The lag terms are influential. Although the impacts of the wind scale fade as time passes, the wind will exert its impact even after four days. After controlling for the wind condition, the effect declines to −5.604.

In addition to wind speed, temperature and humidity also impact air quality. High temperatures can increase oxidation and production of sulfate but reduce nitrate levels through higher volatilization of particles to gas [37]. In Columns (3) and (4), we control for the temperature and rain dummy, respectively. Besides, in Column (5), we control for a full set of meteorological conditions. The ATT is smaller compared to that in Column (1), but it is still significant. The results indicate that the net impact of the COVID-19 lockdown on the AQI is −4.884, or −7.84% compared to what it was in 2019. Hence, our study suggests that news reports and former studies may exaggerate the COVID-19 lockdown’s impact on air pollution by failing to consider meteorological conditions. Our results support the findings of Wang, et al. [38] that severe air pollution is associated with both anthropogenic activities and meteorological conditions.

Notably, the AQI reduction in our results is only half of that estimated in He, Pan, and Tanaka [3]. The major reason for this is that they focused on the AQI in the first-month post-lockdown, while our study covers the full period from the lockdown’s beginning to two weeks after Wuhan‘s reopening. Since their results are based on the first half of the lockdown period in which the most stringent quarantine measures were implemented, their results would overestimate impacts on air pollution.

Ambient air pollution exposure has been found to correlate with respiratory [39,40] and cardiovascular [41,42] diseases, and lead to increased non-trauma deaths [43,44,45]. It has been reported that severe air pollution in China contributes to about 1.6 million premature deaths per year [46]. Pollution control measures can effectively reduce premature deaths effectively even when implemented in a short period [45]. Thus, we project that the decrease of air pollutants reduced the premature deaths by 150,000 nationwide during the research period, according to the all-cause death rate estimated by Dutheil, Baker, and Navel [9]. This number far exceeds the officially reported deaths due to COVID-19.

The overall analysis confirms that the AQI level declined moderately due to the outbreak of COVID-19. However, one may wonder which pollutant level had the most drastic change. Table 3 reports the estimated results of each pollutant. Columns (1)–(6) report the results for SO_2_, NO_2_, CO, O_3_, PM_2.5_, and PM_10_, respectively. The estimation results show diversified impacts of the lockdown measures on different air pollutants, as follows.

First, the average impact on SO_2_ is positively significant at a 99.9 confidence interval. Surprisingly, its concentration during the COVID-19 pandemic increased by 1.68 µg/m^3^, or 14.71%, compared to 2019. This can be partly explained by the extension of the heating season in most northern cities. The statutory heating period ends around March 15 every year. However, in 2020, residents were required to stay at home. Therefore, most local governments postponed the end of collective heating to mid-April. The extended heating season and daily heating time increased SO_2_ emissions due to the massive combustion of coal [47,48].

Second, the concentration of NO_2_ in 2020 decreased by 5.11 µg/m^3^, or 19.24% compared to 2019. NO_2_ can be used to effectively measure traffic intensity, especially in urban areas [12,13]. NO_2_ has been identified as a typical pollutant associated with lockdown measures around the world [9,49,50]. Therefore, on average, vehicle kilometers traveled decreased by roughly 20% during the three months after the lockdown.

Third, CO concentration decreased by 0.105 mg/m^3^, or 30.88%, compared to 2019. Carbon monoxide is a by-product of the incomplete combustion of carbon-containing fuels, and on-road vehicles are a major source of CO in Chinese urban areas [51,52]. Besides, CO pollution mainly comes from small and medium passenger cars, while NO_2_ emissions mainly come from heavy-duty trucks in commercial vehicles. Therefore, as people remained sequestered in their residential areas, there were fewer passenger cars than heavy-duty trucks on the road, which led to a higher reduction of CO than NO_2_.

Fourth, the concentration of ground-level O_3_ increased by 3.881 µg/m^3^, or 3.74%, compared to 2019. O_3_ is formed when nitrogen oxides react with a group of volatile organic compounds (VOCs) under the ultraviolet rays in the presence of sunlight [53]. The increase in the O_3_ level may be a consequence of three combined causes. First, the reduction of NO_x_ changes the ratio of VOCs to NO_x_ in VOC-controlled systems (which applies to most urban areas of China), increasing O_3_ concentration [54,55]. Second, PM_2.5_ reduces atmospheric visibility and significantly blocks ultraviolet rays from sunshine, which further leads to an increase in O_3_ [34]. Third, the reduction of NO_x_ leads to the decrease of nitrogen oxide (NO, NO_x_ = NO_2_ + NO), which further reduces the O_3_ titration (consumption, NO + O_3_ = NO_2_ + O_2_) [54,56,57].

Finally, the concentration of PM_2.5_ decreased by 5.772 µg/m^3^, or 13.75%, compared to 2019. PM_2.5_ is one of the pollutants that most affects air quality and is a secondary pollutant, which is formed in the atmosphere through the reaction, coagulation, or nucleation of precursor gases, especially NO_2_ and SO_2_ [58]. With the reduction of other pollutants, the PM_2.5_ level declined.

Our results can be compared with related studies done in other countries [59]. For example, Sharma, et al. [60] explored the impacts of COVID-19 related restrictions in 20 Indian cities and found that PM_2.5_ had the largest decrease, 43%; by contrast, CO and NO_2_ only decreased by 10% and 18%, respectively, and SO_2_ emissions were negligible. Since both China and India rely largely on coal as their primary energy resource, coal-fired boilers are the highest contributors to SO_2_ and CO emissions. Therefore, the comparison suggests that boilers were more affected by lockdown measures in China, while lockdown impacts on traffic-related emissions were similar in both countries. Tobías et al. [54] also found that NO_2_ emission markedly decreased in Barcelona (Spain), due to the strict restrictions in urban areas. Finally, nearly all studies found significant increases in O_3_ levels [55].

## 4. Discussion: The Dynamic Patterns of the Lockdown Effects

To study the dynamic pattern of how air quality was affected by the COVID-19-related lockdown, we estimated Equation (2) with 12 dummy variables, *post0*, *post1*,…, *post11*, interacting with the *treat* dummy variable. The dummy variable *post0* is taken for the sample period [0, 7) after the event day, while *post1*, …, *post11* are taken for the subsequent 11 weeks.

**Figure 5:**
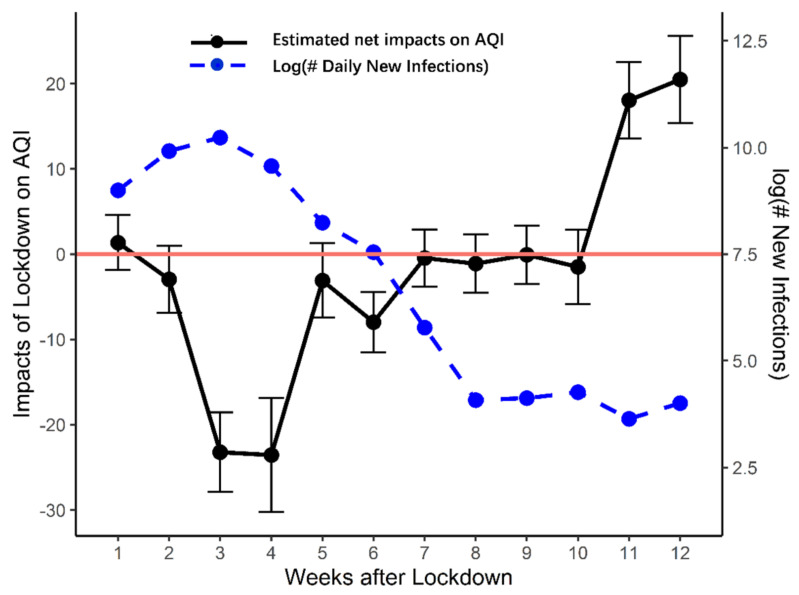
The dynamic air quality index (AQI) response. Note: This figure illustrates the dynamic air quality response. Changes in the daily AQI are the regression coefficients estimated from the DID regression on 12 weeks dummy variables (including *post0*, *post1*, …, *post11*), interacting with a treat dummy variable, which is shown in Equation (2). The week dummy variable *post0* is taken for the sample period [0, 7) after the event day, while *post1*, …, *post11* are taken for the subsequent 11 weeks after the event day. *Treat* is equal to 1 for observations in 2020, and 0 for observations in 2019. The event day is January 23 for 2020 (the date when the Wuhan lockdown was implemented), while the event day for 2019 is February 5, one day before 2019 Chinese New Year’s Eve. The error bar indicates a 95% confidence interval. The incremental # of weekly novel COVID-19 cases is the total number of COVID-19 cases confirmed during the event week.

Figure 5 presents the pattern of the change in AQI. We also illustrate the change in the number of new infections (in its logarithmic form) on the second axis to detect the corresponding impact. In our quasi-DID design, the AQI decrease depicted in Figure 5 is the net impact, which is the air pollution level in 2020, minus that in 2019. Therefore, we observe patterns similar to the mechanism depicted in Figure 1. In the first two weeks of the lockdown, the AQI level is quite the same as it was in 2019, which confirms our assumption that the effects of the lockdown measures and the New Year holiday are comparable. In the third and fourth weeks, as the lockdown continued in most cities, the AQI level dropped by 20 points compared to 2019.

After week 4, the AQI climbed steeply back to its normal level over three weeks. As the number of daily new infections dropped in early March, most cities gradually moved closer to normality; hence the treatment effect fades away. On average, the AQI steadily returned to its normal level about seven weeks after the end of the lockdown of Wuhan city. The temporary improvements in air pollution from the lockdown only lasted for one month for the average city in mainland China.

The turning point comes just one month after the event day. Although most analysts thought that local governors were reluctant to reopen cities due to fear of the epidemic resurging, our analysis shows that the AQI increased immediately after novel infections dropped, which is a quick response. The AQI plateaued for four consecutive weeks after week 7. During this month, the AQI gradually returned to the same level that it was in the same period in 2019 after economic activity resumed. Finally, the AQI increased sharply in the last two weeks. We find strong rebound effects after April 8, immediately after the epicenter lifted its lockdown measures. It suggests that pollution-related industrial and business activities bounced back after 2019 as the government called for a restart of the economy.

**Figure 6:**
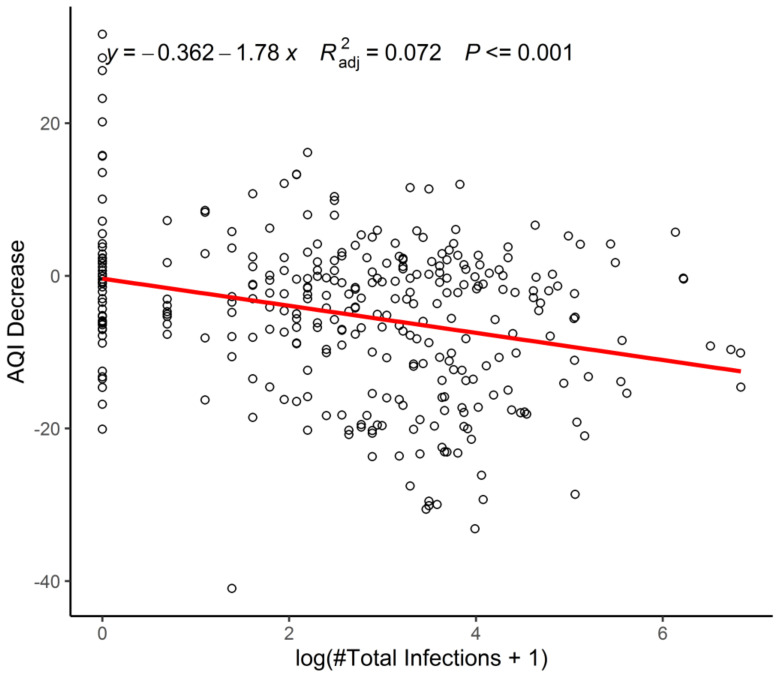
The association between the air quality index (AQI) and the daily new infections. Note: This figure shows the impact of the daily new COVID-19 cases on the AQI across cities. It displays the simple scatterplot between the AQI and the total number of COVID-19 cases as of 22 April 2020. We also included the fitted line in the scatterplot.

Figure 6 depicts the association between the AQI and daily new infections. The straight line in the scatterplot shows that the more daily new infections, the better the air quality. When the number of daily new infections is highest, human activities are strictly monitored, and people are required to stay at home. As the number of daily new infections gradually decreases, control measures also gradually relax. Simultaneously, people start to resume work, and companies begin production activities, leading to a decline in air quality.

To summarize, our estimation suggests that the lockdown’s impact on the AQI persisted for nearly four weeks, from January 23 to late February. The AQI then returned to its normal level and seesawed up and down for more than one month. Lastly, as daily new infections went down and mass quarantine measures were relaxed and then eventually removed, and air pollution increased significantly.

**Figure 7:**
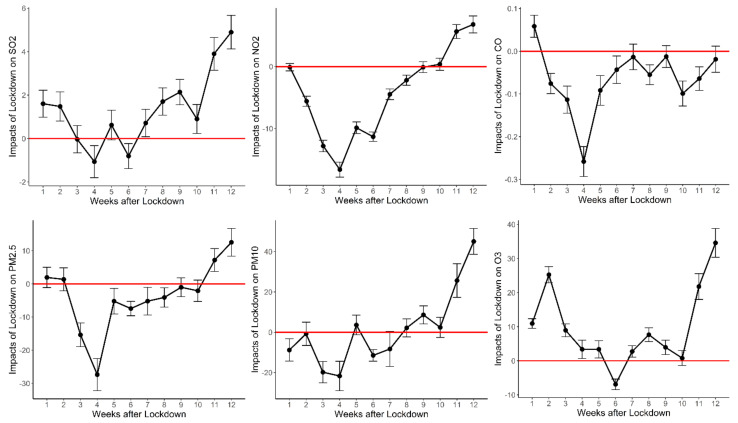
Concentration changes of air pollutants over time: by categories. Note: This figure presents the dynamic pollutant concentration responses by categories. Changes in daily average concentration are the regression coefficients estimated from the DID regression on 12 week dummy variables (including *post0*, *post1*, …, *post11*), interacting with a treat dummy variable, which is shown in Equation (2). The week dummy variable *post0* is taken for the sample period [0, 7) after the event day, while *post1*, …, *post11* are taken for the subsequent 11 weeks after the event day. *Treat* is equal to 1 for observations in 2020, and 0 for observations in 2019. The event day is January 23 for 2020 (the date when the Wuhan lockdown was implemented), while the event day for 2019 is February 5, one day before the 2019 Chinese New Year’s Eve. The error bar indicates a 95% confidence interval.

Figure 7 shows the dynamic change of each major air pollutant’s concentration. Although their u-shape patterns are similar to that of the AQI, we find subtle differences among pollutants, which are worth exploring. However, SO_2_ exhibits a unique pattern during the epidemic period. This is because most northern cities expanded their heating season to mid-April, which increased SO_2_ emissions.

Among the six pollutant criteria, the changes in NO_2_ levels are the most typical. In urban areas, vehicles are the major source of NO_2_ emission. Therefore, NO_2_ concentrations are an effective way to measure traffic. As shown in Figure 7, in the first week of lockdown, NO_2_ concentration is similar to that in 2019. However, soon after, there is a steep decline in NO_2_ levels, which suggests that residents were still sequestered in their houses. It only started to increase after week 4.

The changing trend of PM_2.5_ mirrors that of NO_2_. In the first two weeks of lockdown, the PM_2.5_ level was higher than that of the same period in 2019, and then it plummeted. It started to bounce back after the fourth week. However, it remained lower than the PM_2.5_ levels of the same period in 2019. In the eleventh week, it returned to a level higher than that in 2019. The overall changing trend of PM_10_ also declined first and then increased. For PM_10_, its level in the first week during the lockdown was lower than that of the same period in 2019, while in the second week, it rose to the same level as in 2019, then began to decline. It started to bounce back after the fourth week. However, there was a decline from the fifth to the sixth week. Again, it continued to rise, and after eight weeks, it reached a level that was higher than that of the same period in 2019. These patterns can be explained as follows. The main sources of PM_2.5_ are the residues of power generation, industrial production, vehicle exhaust emissions, and coal-burning [60]. Although power generation did not decrease, and coal-burning increased, vehicle exhaust emissions and industrial production greatly reduced, thus resulting in a reduction of PM_2.5_ during the lockdown. However, most of the time, the PM_10_ level was higher than or equal to that of the same period in 2019, which is most likely due to increased coal-burning during the lockdown. Meanwhile, the reduction of certain pollutants in the atmosphere changed the composition ratio of substances, allowing compounds to interact to form more fine particles [6].

Regarding O_3_, its level rose from the first to the second week and then plummeted. However, it was not until the sixth week that for the first time, its level fell below that of the same period in 2019. It then started to bounce back, and it remained higher than that of the same period in 2019. The increase of O_3_ can be explained by three reasons. First, the decrease in NO_x_ led to the change in its ratio to VOCs, which in turn led to an increase in O_3_ concentration [55,61]. Second, the lower PM_2.5_ concentrations reduced the scattering and absorption of sunlight, which increased UV radiation and led to a higher O_3_ concentration [34]. Third, the decreased NO_x_ reduced the consumption of O_3_ (NO + O_3_ = NO_2_ + O_2_) in urban areas, thus leading to higher O_3_ concentrations [50].

To sum up, the heterogeneous dynamic patterns of different pollutants are related to changes in substantive human activities. Significant rebound effects were detected for almost all pollutants after the lockdown was lifted, which triggers concerns about the long-term impacts of the COVID-19 lockdown on air pollution.

**Figure 8:**
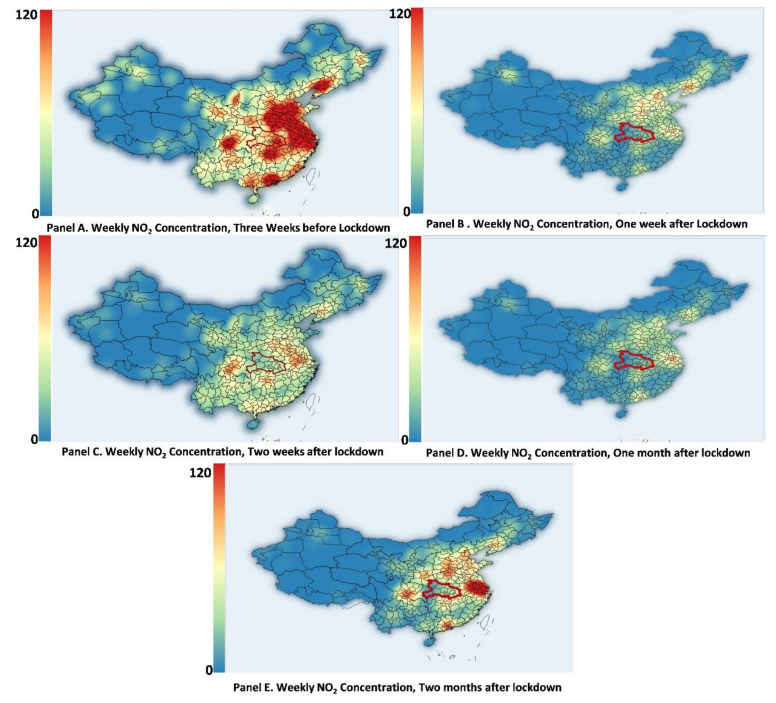
Heat maps of weekly NO_2_ concentration across China during the COVID-19-related lockdown period.Notes: This figure presents the weekly average NO_2_ concentration across China before and after the COVID-19 lockdown. The epicenter, Hubei Province is outlined in a red circle. The weekly average is adopted to curb the stochastic influence of weather conditions.

Although the above results indicate a quick rebound on average, there is much heterogeneity among regions. Figure 8 depicts a heat map of urban NO_2_ concentration nationwide, which shows the spatial-temporal fluctuation of NO_2_ concentration. In Panel A, NO_2_ concentration three weeks pre-lockdown can be considered as the usual pattern. Four pollution hotspots are clear and include the Capital Region, the Yangtze River Delta, the Pearl River Delta, and the Sichuan Basin. Compared with Panel A, Panel B indicates that the lockdown reduced NO_2_ concentrations drastically and uniformly from the first to the fourth week nationwide. However, as shown in Panel E, air pollution rebounded much faster in the Yangtze River Delta and the Pearl River Delta and was much slower to increase in other hotspots, namely the Sichuan Basin and the Capital Region. The stark differences in rebound suggest regional differences in industrial structures and other institutional factors. For example, the export sectors in the southern regions are more vital, especially those manufacturing personal protective equipment, which was affected by the surge of infections in other countries.

## 5. Conclusions

In this study, we conducted a quasi-DID analysis of the impacts of COVID-19-related lockdown measures on air quality in China. Our study covers 367 prefectural- and county-level cities during the epidemic period from the beginning of the lockdown until two weeks after its lifting in Wuhan. The results suggest the following.

First, on average, the AQI decreased by about 7%. Although our results indicate immense improvements, air quality levels were still over the threshold set by the WHO and Chinese standards. Second, we detected significant heterogeneous impacts on different pollutants. CO had the biggest drop, about 30%, and NO_2_ had the second-largest drop, about 20%. In contrast, O_3_ increased by 3.74%. We attribute these differences to the lockdown’s heterogeneous impacts on different anthropogenic activities. Concentrations of CO and NO_2_ were sharply reduced from traffic restriction measures meant to contain the viral transmission, while O_3_ increased because the reduction of PM_2.5_ and PM_10_ in the troposphere increased the UV radiation, which in turn increased photochemical reaction intensity. Third, although the AQI reduced steeply after the lockdown, it increased immediately after the number of novel infections dropped, which is a quick response. Finally, we also detected preliminary cues of the rebound effect, immediately after the lifting of lockdown measures in Wuhan.

Our study also sheds some light on the effectiveness of the quick-response measures put into place after the declaration of an environmental emergency, especially when urban air quality reaches the red alert level, which, according to WHO standards, is extremely toxic for humans. Quick and temporary restrictive measures, including activity suspension of heavy-polluting plants and traffic restrictions based on the last digit of license plate numbers, can be effective at lowering NO_2_, SO_2_, and PM_2.5_ concentrations. However, policymakers should be cautious about increases in O_3_ concentrations.

One limitation of our study is that as the epidemic is fading away in China, its long-term impacts are still not clear. On the one hand, some suggest that environmental degradation due to the extreme, massive economic stimulus will occur. On the other hand, COVID-19 is more infectious compared to severe acute respiratory syndrome (SARS), which emerged in 2002 in China. Lifestyles may change permanently in a more sustainable direction. For example, virtual meetings are now held more frequently, and white-collar workers prefer working from home. Moreover, instead of simply turning to the old playbook of investment stimulus, the government has launched a new infrastructure initiative, which mainly incorporates fifth-generation networks, industrial internet, inter-city transit systems, vehicle charging stations, data centers, and several other projects. These policies would lead to more sustainable growth. Therefore, instead of focusing on the short-term environmental effects related to the lockdown, it would be worthwhile to expand our research to explore the potential permanent environmental impacts of the COVID-19 lockdown.

## Figures and Tables

**Table 1:** Summary Statistics of the Urban Ambient Air Quality Index (AQI).

Panel A: 2019 Sample							
	(1)	(2)	(3)	(4)	(5)	(6)	(7)
	Obs.	Mean	p10	p25	p50	p75	p90
All	45718	77.32	36.33	47.58	64.21	89.63	134.85
pre: [−22, −1]	7944	92.40	41.46	55.75	78.94	113.88	158.82
post: [0, 93]	33444	71.55	35.92	46.46	61.08	81.42	116.79
Panel B: 2020 Sample							
	(1)	(2)	(3)	(4)	(5)	(6)	(7)
	Obs.	Mean	p10	p25	p50	p75	p90
All	41960	67.86	27.96	39.21	56.58	79.05	116.71
pre: [−22, −1]	7955	90.25	31.90	46.96	72.71	116.94	179.36
post: [0, 93]	33644	62.32	27.38	37.92	54.25	73.46	99.28
Panel C: Mean difference of city-level air pollutants							
	(1)	(2)	(3)		(1)	(2)	(3)
	pre:2019	post:2019	post-pre:2019		pre:2020	post:2020	post-pre:2020
AQI	92.40	71.55	−20.85 ***		90.25	62.32	−27.92 ***
***Type:***							
SO_2_	16.42	11.42	−5.00 ***		14.21	10.48	−3.73 ***
NO_2_	36.36	26.56	−9.80 ***		37.24	22.21	−15.03 ***
CO	1.15	0.80	−0.34 ***		1.15	0.74	−0.41 ***
O_3_	75.31	103.77	28.46 ***		69.24	100.84	31.6 ***
PM_2.5_	63.43	41.97	−21.45 ***		65.18	38.52	−26.66 ***
PM_10_	99.53	76.79	−22.73 ***		86.60	66.58	−20.02 ***

Note: The summary statistics are calculated for the daily AQI of all cities in the sample. The unit for CO is mg per cubic meter, and the unit for other pollutants is µg per cubic meter, both under standard conditions. Day zero is January 23 for 2020 (the date when the Wuhan lockdown was implemented), while day zero for 2019 is February 5. The pre-period is defined as [−22, −1], while the post-period is defined as [0, 93], according to day zero. *** *p* < 0.01, ** *p* < 0.05, * *p* < 0.1.

**Table 2:** The impact of the COVID-19 lockdown on ambient air quality.

Dependent Variable	AQI				
	(1)	(2)	(3)	(4)	(5)
Treat*Post	−7.125 ***	−5.604 ***	−4.749 **	−5.831 ***	−4.884 **
	(1.605)	(1.842)	(1.945)	(1.827)	(1.944)
Treat	−1.412	−4.176 ***	−4.226 ***	−4.034 **	−4.172 ***
	(1.452)	(1.598)	(1.596)	(1.588)	(1.602)
Wind Speed		−0.424	−0.559 *	−0.372	−0.478
		(0.296)	(0.292)	(0.296)	(0.293)
L.Wind Speed		−6.094 ***	−6.033 ***	−6.088 ***	−6.072 ***
		(0.461)	(0.462)	(0.460)	(0.459)
L2. Wind Speed		−5.146 ***	−5.172 ***	−5.151 ***	−5.163 ***
		(0.311)	(0.309)	(0.311)	(0.307)
L3. Wind Speed		−2.759 ***	−2.799 ***	−2.763 ***	−2.865 ***
		(0.286)	(0.280)	(0.286)	(0.283)
L4. Wind Speed		−2.037 ***	−2.199 ***	−2.054 ***	−2.219 ***
		(0.314)	(0.294)	(0.314)	(0.293)
Temperature (Minimum)			0.093		0.120
			(0.098)		(0.110)
Temperature (Highest)			0.385 **		0.396 **
			(0.166)		(0.178)
Sunny				1.189 ***	1.296 ***
				(0.433)	(0.487)
Constant	100.713 ***	112.625 ***	112.971 ***	111.668 ***	117.206 ***
	(1.937)	(3.152)	(3.078)	(3.173)	(4.102)
Date Dummy	Y	Y	Y	Y	Y
City Dummy	Y	Y	Y	Y	Y
Groups	367	335	335	335	335
Sample	83,710	71,597	71,597	71,597	71,597
adj R2	0.127	0.141	0.143	0.141	0.144

Note: This table reports the regression results of the average impact of the COVID-19 lockdown on the Air Quality Index (AQI) of all cities in the sample. The dependent variable is the AQI of each city. The dummy variable “treat” is defined as 1 for observations in 2020, and 0 otherwise. “Post” is defined as 1 for the post periods [0, 58], and 0 otherwise. The event day is defined as January 23 for 2020 (the date on which the Wuhan lockdown was implemented), while the event day for 2019 is defined as February 5. Standard errors reported in parentheses are clustered at the city level. *** *p* < 0.01, ** *p* < 0.05, * *p* < 0.1.

**Table 3:** The impact of the COVID-19 lockdown on different air pollutants.

	(1)	(2)	(3)	(4)	(5)	(6)
	SO_2_ Concentration	NO_2_ Concentration	CO Concentration	O_3_ Concentration	PM_2.5_ Concentration	PM_10_ Concentration
Treat*Post	1.679 ***	−5.113 ***	−0.105 ***	3.881 ***	−5.772 ***	2.721
	(0.343)	(0.402)	(0.012)	(0.794)	(1.526)	(2.198)
Treat	−3.073 ***	−0.156	0.019	−3.134 ***	1.077	−11.821 ***
	(0.425)	(0.427)	(0.013)	(0.605)	(1.347)	(1.647)
Wind Speed	−0.149 **	−0.120 *	−0.005 **	1.945 ***	−0.645 ***	0.725 *
	(0.067)	(0.065)	(0.002)	(0.190)	(0.209)	(0.387)
L.Wind Speed	−1.391 ***	−4.008 ***	−0.075 ***	0.584 ***	−5.363 ***	−0.563
	(0.106)	(0.113)	(0.004)	(0.177)	(0.294)	(0.645)
L2. Wind Speed	−1.239 ***	−3.363 ***	−0.084 ***	−2.013 ***	−7.452 ***	−6.733 ***
	(0.094)	(0.105)	(0.004)	(0.183)	(0.346)	(0.476)
L3. Wind Speed	−0.458 ***	−0.879 ***	−0.038 ***	−2.036 ***	−3.954 ***	−4.282 ***
	(0.056)	(0.069)	(0.003)	(0.173)	(0.295)	(0.428)
Temperature (Minimum)	0.129 ***	0.270 ***	−0.003 ***	2.105 ***	−0.242 ***	0.179
	(0.020)	(0.021)	(0.001)	(0.076)	(0.087)	(0.139)
Temperature (Highest)	−0.241 ***	−0.362 ***	−0.002 *	−0.835 ***	0.314 **	0.649 **
	(0.027)	(0.031)	(0.001)	(0.081)	(0.151)	(0.304)
No-rain	−0.322 ***	−0.735 ***	−0.029 ***	0.234	−0.735 **	0.711
	(0.076)	(0.101)	(0.003)	(0.329)	(0.351)	(0.662)
Constant	22.881 ***	54.813 ***	1.648 ***	57.874 ***	108.913 ***	135.890 ***
	(1.059)	(1.119)	(0.040)	(2.182)	(3.177)	(5.310)
Groups	335	335	335	335	335	335
Sample	72,281	72,281	72,281	72,281	72,281	72,281
adj R2	0.153	0.403	0.347	0.418	0.208	0.059

Note: This table reports the regression results of the average impact of the COVID-19 lockdown on each pollutant for all cities in the sample. Standard errors reported in parentheses are clustered at the city level. *** *p* < 0.01, ** *p* < 0.05, * *p* < 0.1.

## Data Availability

The data presented in this study are available on request from the corresponding author. The urban air quality data can also be downloaded directly from website of China National Urban Air Quality Real-time Publishing Platform (http://106.37.208.233:20035/) and the prefectural COVID infection data can be collected from the https://github.com/GuangchuangYu/nCov2019.
